# Characteristics and outcome in cardiogenic shock according to vascular access site for percutaneous coronary intervention

**DOI:** 10.1093/ehjacc/zuae078

**Published:** 2024-06-26

**Authors:** Elma J Peters, Margriet Bogerd, Sanne Ten Berg, Marijke J C Timmermans, Annemarie E Engström, Holger Thiele, Christian Jung, Benedikt Schrage, Krischan D Sjauw, Niels J W Verouden, Koen Teeuwen, Admir Dedic, Martijn Meuwissen, Peter W Danse, Bimmer E P M Claessen, José P S Henriques, Participating Centers of the PCI Registration Committee of the Netherlands Heart Registration, M Meuwissen, J P Henriques, K M J Marques, K Teeuwen, J Daemen, C E Schotborgh, R J R Snijder, R Scherptong, J Brouwer, A Dedic, C Camaro, P W Danse, E Lipšic, A O Kraaijeveld

**Affiliations:** Department of Cardiology, Heart Center, Amsterdam University Medical Centre, Amsterdam Cardiovascular Sciences, Meibergdreef 9, 1105 AZ Amsterdam, The Netherlands; Department of Cardiology, Heart Center, Amsterdam University Medical Centre, Amsterdam Cardiovascular Sciences, Meibergdreef 9, 1105 AZ Amsterdam, The Netherlands; Department of Cardiology, Heart Center, Amsterdam University Medical Centre, Amsterdam Cardiovascular Sciences, Meibergdreef 9, 1105 AZ Amsterdam, The Netherlands; Netherlands Heart Registration, Utrecht, The Netherlands; Department of Intensive Care, Amsterdam University Medical Centre, Amsterdam, The Netherlands; Department of Internal Medicine/Cardiology, Heart Centre Leipzig, University of Leipzig and Leipzig Heart Science, Leipzig, Germany; Department of Cardiology, Pulmonology and Vascular Medicine, Medical Faculty, Heinrich-Heine-University Duesseldorf, Duesseldorf, Germany; Department of Cardiology, University Heart and Vascular Centre Hamburg, Hamburg, Germany; Department of Cardiology, St. Antonius Hospital, Nieuwegein, The Netherlands; Department of Cardiology, Heart Center, Amsterdam University Medical Centre, Amsterdam Cardiovascular Sciences, Meibergdreef 9, 1105 AZ Amsterdam, The Netherlands; Heart Center, Department of Interventional Cardiology, Catharina Hospital Eindhoven, Eindhoven, The Netherlands; Department of Cardiology, Northwest Clinics, Alkmaar, The Netherlands; Department of Cardiology, Amphia Hospital, Breda, The Netherlands; Department of Cardiology, Rijnstate Hospital, Arnhem, The Netherlands; Department of Cardiology, Heart Center, Amsterdam University Medical Centre, Amsterdam Cardiovascular Sciences, Meibergdreef 9, 1105 AZ Amsterdam, The Netherlands; Department of Cardiology, Heart Center, Amsterdam University Medical Centre, Amsterdam Cardiovascular Sciences, Meibergdreef 9, 1105 AZ Amsterdam, The Netherlands

**Keywords:** Cardiogenic shock, Vascular access, Myocardial infarction, PCI, Femoral artery, Radial artery, Revascularization

## Abstract

**Aims:**

The optimal vascular access site for percutaneous coronary interventions (PCIs) in patients with acute myocardial infarction (AMI) complicated by cardiogenic shock (CS) remains uncertain. While observational data favour transradial access (TRA) due to lower complication rates and mortality, transfemoral access (TFA) PCI offers advantages such as shorter access and procedure times, along with quicker escalation to mechanical circulatory support (MCS). In this study, we aimed to investigate factors associated with a transfemoral approach and compare mortality rates between TRA and TFA in AMI-CS patients undergoing PCI.

**Methods and results:**

Data from a nationwide registry of AMI-CS patients undergoing PCI (2017–2021) were analysed. We compared patient demographics, procedural details, and outcomes between TRA and TFA groups. Logistic regression identified access site factors and radial-to-femoral crossover predictors. Propensity score–matched (PSM) analysis examined the impact of access site on mortality. Of the 1562 patients, 45% underwent TRA PCI, with an increasing trend over time. Transfemoral access patients were more often female, had a history of coronary artery bypass grafting, lower blood pressure, higher resuscitation and intubation rates, and elevated lactate levels. After PSM, 30-day mortality was lower in TRA (33% vs. 46%, *P* < 0.001). Predictors for crossover included left coronary artery interventions, multivessel PCI, and MCS initiation.

**Conclusion:**

Significant differences exist between TRA and TFA PCI in AMI-CS. Transfemoral access was more common in patients with worse haemodynamics and was associated with higher 30-day mortality compared with TRA. This mortality difference persisted in the PSM analysis.

## Graphical Abstract

**Graphical Abstract:**
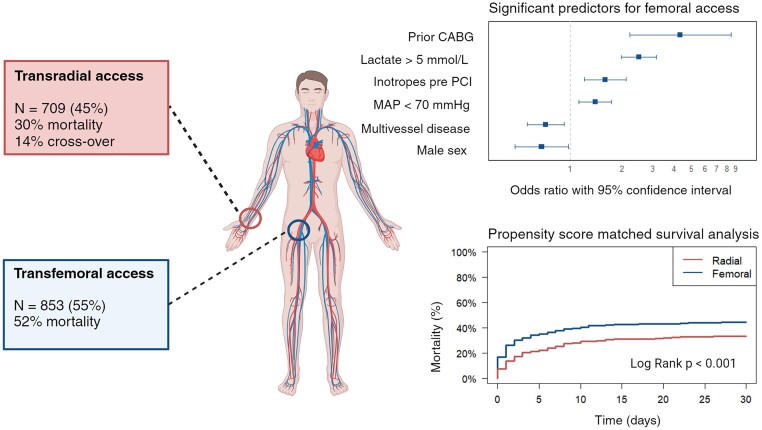
Study cohort; predictors for femoral access and propensity score–matched survival analysis. CABG, coronary artery bypass grafting; MAP, mean arterial pressure. Created with BioRender.com

## Introduction

Percutaneous coronary intervention (PCI) has traditionally predominantly been performed via the transfemoral access (TFA) route. However, radial access has gained momentum since its description as an alternative technique in 1993.^1^ Specific anatomical advantages of the radial artery, such as its superficial location which conveniently allows for easy and effective compression, result in significantly fewer vascular complications and access site bleeding compared with the femoral artery. The reduction in access site complications, in turn, manifested as a decrease in acute kidney injury and even mortality compared with TFA.^2,3^ Transradial access (TRA) therefore became the preferred and recommended approach for PCI, in particular for patients with acute myocardial infarction (AMI).^4,5^

Limited randomized evidence is available on the treatment of AMI that is complicated by cardiogenic shock (CS), but since the results of the SHOCK trial have been published in 1999, early revascularization has established its place in all treatment guidelines.^6–8^ However, there is an ongoing debate on the optimal vascular approach in these critically ill patients. It has been repeatedly suggested that also in CS, TRA is associated with a reduction in complications.^9^ Unfortunately, all studies aiming to compare outcomes between the two vascular access sites are subject to confounding by indication as operators may tend to choose TFA more often in sicker patients. Reasons for this include the possibility for upscaling to large-bore access to facilitate mechanical circulatory support (MCS) insertion and the fact that the femoral artery may be easier to identify than the radial artery, especially in patients with low blood pressures.

To address this issue, we analysed data from the Netherlands Heart Registration (NHR) where the access site is provided in PCI procedures for CS. We aimed to provide insights into patient characteristics and clinical outcome in CS patients undergoing PCI through either TRA or TFA. Furthermore, we investigated the temporal trend in choice of access site and the factors that are associated with radial-to-femoral crossover during the procedure.

## Methods

### Data source, endpoints, and follow-up

The NHR is a nationwide registry in which patient-, procedural- and outcome-level data on all PCIs performed in the Netherlands are prospectively registered.^10^ Data collection is performed up to high-quality standards.^11^ In 14 Dutch hospitals, additional data were retrospectively collected in patients with CS undergoing PCI. This process has previously been described in detail elsewhere.^12^ No ethical approval was required under the Medical Research Involving Human Subjects Act (WMO) as was confirmed by the Medical Research Ethics Committees United (MEC-U). Information on mortality at 30 days and 1 year was collected using the Dutch Personal Records Database (Statistics Netherlands, The Hague, The Netherlands).

### Study population

Eligible patients underwent PCI between January 2017 and September 2021 in 1 of the 14 hospitals of the NHR CS registry. Patients were included in the current study if they fulfilled the NHR criteria of CS, where CS is defined as follows: (i) the presence of hypotension [systolic blood pressure (SBP) ≤ 90 mmHg for ≥30 min or support to maintain SBP ≥ 90 mmHg) and (ii) end-organ hypoperfusion (cold extremities and/or oliguria <30 mL/h and/or tachycardia ≥60 b.p.m.). Patients were excluded from the analysis if (i) the arterial access site for PCI was unknown, or other than femoral or radial, or if (ii) shock symptoms developed during or after PCI but not prior to the start of the procedure. All procedural steps, including revascularization strategy, anti-coagulation, and anti-thrombotic therapy, were carried out in accordance with local protocols.

### Statistical analysis

The baseline patient- and procedure-level characteristics and mortality rates were compared between TRA and TFA procedures. Categorical data were presented as numbers/total numbers and percentages and compared using the *χ*^2^ test. Numerical data were displayed as medians with interquartile range (IQR) and compared using the Mann–Whitney U test or as means with standard deviation and compared with a *t*-test, depending on normality.

Univariate logistic regression analyses were performed to investigate the association between patient characteristics and choice of vascular access site and crossover. All explanatory variables from *Table 1* were considered for the regression analysis but only included in the multivariable model if univariate regression was significant at a *P* level of 0.10. No multivariable logistic regression was done for the crossover analysis due to the relatively small number of events.

**Table 1:** Baseline characteristics of all patients with shock pre-percutaneous coronary intervention

	All (*N* = 1562)	Radial access (*N* = 709)	Femoral access (*N* = 853)	*P*-value
Baseline and medical history				
Age (years)	67 (58–75)	67 (58–74)	67 (58–76)	0.302
Male sex, *n* (%)	1150 (74)	551 (78)	599 (70)	<0.001
BMI (kg/m^2^)	26 (24–29)	26 (24–29)	26 (24–29)	0.929
Height (cm)	175 (168–180)	175 (170–180)	175 (167–180)	0.023
Medical history, *n*/*N* (%)				
Diabetes	324/1480 (22)	136/687 (20)	188/793 (24)	0.070
Multivessel disease	916/1554 (59)	435/706 (62)	481/848 (57)	0.051
Prior CABG	96/1536 (6)	17/703 (2)	79/833 (10)	<0.001
Prior MI	324/1510 (21)	134/695 (19)	190/815 (23)	0.057
Prior PCI	267/1514 (18)	114/698 (16)	153/816 (19)	0.219
Current presentation				
MAP (mmHg)	71 (58–89)	73 (61–91)	70 (55–87)	<0.001
Heart rate (b.p.m.)	83 (61–104)	82 (63–105)	85 (60–103)	0.702
SOFA score	10 (8–12)	10 (7–12)	10 (8–13)	0.054
No. of inotropes, *n*/*N* (%)				
0	627/1494 (42)	366/687 (53)	261/807 (32)	<0.001
1	456/1494 (31)	173/687 (25)	283/807 (35)	<0.001
2	325/1494 (22)	115/687 (17)	210/807 (26)	<0.001
≥3	86/1494 (7)	33/687 (5)	53/807 (7)	0.145
Aetiology, *n*/*N* (%)				
STEMI	1314/1550 (85)	587/703 (83)	727/847 (86)	0.203
NSTEMI	208/1550 (13)	102/703 (15)	106/847 (13)	0.251
Symptoms > 24 h	231/1347 (17)	96/628 (15)	135/719 (19)	0.090
Resuscitated	794/1556 (51)	315/707 (45)	479/849 (56)	<0.001
Intubated	786/1557 (51)	287/706 (41)	499/851 (59)	<0.001
Laboratory values				
Glucose (mmol/L)	12.4 (9.2–17.4)	11.3 (8.7–15.5)	13.5 (9.8–19.1)	<0.001
Lactate (mmol/L)	6.0 (2.8–9.8)	4.4 (2.3–8.0)	6.8 (3.5–11)	<0.001
Haemoglobin (mmol/L)	8.4 (7.3–9.2)	8.5 (7.5–9.3)	8.2 (7.2–9.1)	0.002
eGFR (mL/min)	60 (47–74)	63 (49–78)	58 (45–70)	<0.001
Mechanical circulatory support, *n*/*N* (%)				
None	1143/1513 (76)	533/676 (79)	610/837 (73)	0.007
Before PCI	161/1513 (11)	59/676 (9)	102/837 (12)	0.030
In cath lab, after PCI	174/1513 (12)	69/676 (10)	105/837 (13)	0.156
Outcome				
30-day mortality, *n*/*N* (%)	650/1550 (42)	209/702 (30)	441/848 (52)	<0.001
1-year mortality, *n*/*N* (%)	500/1076 (46)	176/487 (36)	324/589 (55)	<0.001

Values are median (interquartile range) unless indicated otherwise. Inotropes included the following agents initiated prior to PCI: noradrenaline, adrenaline, dobutamine, dopamine, and milrinone/enoximone. Resuscitated, either in or out of hospital cardiac arrest pre-PCI.

BMI, body mass index; CABG, coronary artery bypass grafting; MI, myocardial infarction; PCI, percutaneous coronary intervention; MAP, mean arterial pressure; b.p.m., beats per minute; STEMI, ST-elevation myocardial infarction; NSTEMI, non-ST-elevation myocardial infarction.

For the crossover analysis, continuous variables were dichotomized using the point closest to the upper left corner of the receiver operating characteristic (ROC) curve as the cut-off.^13^ Crossover was defined as the transition from radial to femoral access within the same procedure. To ensure maximal power, all regression analyses were done on multiple imputed data. Five imputed data sets were generated by means of predictive mean matching to account for missing data.

Survival was analysed at 30 days and 1 year using the Kaplan–Meier method and compared between TRA and TFA using the log-rank statistic. A propensity score–matched (PSM) analysis was performed to investigate the impact of access site on 30-day mortality. Propensity scores were calculated using all available patient-level characteristics. Calculations were done in five multiple imputed data sets using the nearest method with a calliper of 0.10. Subsequently, the propensity scores were remerged into the data set with missing data, and TRA patients were matched to TFA patients in a 1:1 ratio. This resulted in 507 matches.

A sensitivity analysis for patients who did not receive any form of MCS before or during their stay in the catheterization laboratory was done for the baseline characteristics. An additional analysis of baseline characteristics was performed including patients who developed CS during or directly after the PCI procedure as these patients may in fact already be in some state of shock.

Furthermore, temporal trends were analysed using the Mann–Kendall test. The year 2017 was excluded from the temporary trend analysis due to incomplete registration of the primary access site in this year. Additionally, the crossover analysis only included patients treated from 2019 onwards since the registration of a second access route was not mandatory in 2017 and 2018.

Baseline and regression analyses were performed with IBM SPSS Statistics version 28.0 (IBM corporation, Armonk, NY, USA). The PSM analysis was performed using R (2022, Vienna, Austria). All R packages utilized in this study are detailed in the online supplement, with packages mice (v3.15; Van Buuren and Groothuis-Oudshoorn 2011), MatchThem (v1.1; Pihgar *et al.* 2021), and MatchIt (v4.5.5; Ho *et al.* 2011).

## Results

### Baseline

A total of 2328 patients were identified who had CS and underwent PCI between January 2017 and September 2021. Of these, 283 patients were excluded from the analysis because the primary vascular access site was either unknown or other than radial or femoral. We specifically investigated the differences between TRA and TFA in patients with CS before PCI. For the primary analysis, we therefore excluded patients who developed shock during or after the PCI procedure (*n* = 483). Consequently, this cohort consists of 1562 patients with CS before PCI.

As shown in *Table 1*, the median age in the entire cohort was 67 (IQR 58–75) years, and 1150 patients (74%) were male. The mean arterial pressure upon arrival in the hospital was 71 mmHg with a median systolic pressure of 94 mmHg. Roughly 60% of patients were treated with at least one inotrope or vasopressor. Of all patients, 324 (22%) had a history of diabetes and 916 (59%) had multivessel coronary artery disease. Furthermore, 794 patients (51%) were resuscitated prior to the PCI.

### Access site

The primary vascular access site was femoral in 853 of patients (55%) and radial in 709 patients (45%). Whereas TRA was used in 39% of AMI-CS PCIs in 2018, a significant increase was seen to 47% in 2021 (*P* = 0.04). In 77 patients (14%) with initial radial arterial access, crossover to the femoral artery occurred during the procedure. Patients who underwent TFA PCI differed from the TRA patients in several aspects. In TFA patients, a history of coronary artery bypass grafting (CABG) was more prevalent (10% vs. 2%, *P* < 0.001), and the hospital presentation was more often preceded by a cardiac arrest (56% vs. 45%, *P* < 0.001). In general, TFA patients presented with worse haemodynamics: they had a lower blood pressure on presentation and increased levels of markers of organ hypoperfusion such as lactate and creatinine. The rate of vasoactive medication use before the PCI was also significantly higher than in the TRA group (68% vs. 47%, *P* < 0.001) that received at least one inotrope or vasopressor before PCI. Multivariate logistic regression revealed multiple factors that were significantly associated with the choice for TFA after correction for relevant confounders. These factors included sex, a history of CABG, presence of multivessel disease, a lower MAP, a higher blood lactate level, and the administration of inotropes before PCI (see *Table 2*). These results persisted when patients that developed shock during or after PCI were added to the analysis (see Supplementary material online, *Table S1 a*nd *Table S2*). The distribution of patient characteristics and comorbidities showed a similar pattern even though the proportion of patients treated by TRA (1013/2045, 50%) was slightly increased in this sensitivity analysis.

**Table 2:** Univariate and multivariate logistic regression for femoral access in all patients with shock pre-percutaneous coronary intervention

	OR univariate	95% CI	*P*-value	OR multivariate	95% CI	*P*-value
Year (reference 2021)						
2020	0.973	0.736–1.287	0.848	0.943	0.683–1.302	0.720
2019	1.095	0.821–1.460	0.538	1.120	0.803–1.562	0.505
2018	1.384	1.010–1.8698	0.043	1.321	0.921–1.894	0.131
2017	0.736	0.451–1.203	0.219	0.515	0.263–1.008	0.053
Age	1.004	0.996–1.012	0.378			
Male sex	0.674	0.536–0.848	<0.001	0.681	0.477–0.972	0.034
BMI	1.005	0.991–1.020	0.449			
Height	0.984	0.974–0.995	0.003	0.988	0.971–1.004	0.143
Diabetes mellitus	1.262	0.982–1.623	0.069	0.907	0.663–1.241	0.541
Prior CABG	4.053	2.335–7.034	<0.001	4.227	2.158–8.280	<0.001
Prior MI	1.277	0.999–1.631	0.051	1.301	0.957–1.769	0.093
Prior PCI	1.188	0.911–1.550	0.203			
Multivessel disease	0.823	0.671–1.010	0.062	0.730	0.572–0.930	0.011
Intubated pre-PCI	2.058	1.681–2.519	<0.001	1.330	0.916–1.931	0.134
Resuscitated	1.611	1.317–1.970	<0.001	1.033	0.730–1.462	0.852
STEMI (vs. NSTEMI)	1.226	0.916–1.642	0.170			
MAP	0.993	0.988–0.997	<0.001	0.992	0.987–0.997	0.003
Heart rate	0.999	0.995–1.003	0.690			
SOFA score	1.099	1.040–1.161	0.003	1.049	0.972–1.132	0.194
Inotropes pre (yes/no)	2.314	1.889–2.835	<0.001	1.466	1.098–1.958	0.010
Duration of symptoms						
<3 h	0.946	0.755–1.187	0.629			
>24 h	1.271	0.964–1.671	0.089	1.214	0.852–1.732	0.282
Lactate	1.128	1.100–1.156	<0.001	1.087	1.052–1.123	<0.001
Glucose	1.062	1.044–1.081	<0.001	1.015	0.991–1.040	0.217
Haemoglobin	0.897	0.836–0.963	0.003	0.947	0.861–1.041	0.259
eGFR	0.991	0.987–0.995	<0.001	0.996	0.996–1.000	0.073
MCS before PCI	1.439	1.009–2.052	0.044	1.062	0.684–1.649	0.787

BMI per kg/m^2^; height per cm; MAP per mmHg; SOFA per point; lactate per mmol/L; glucose per mmol/L; haemoglobin per mmol/L; eGFR per mL/min.

BMI, body mass index; CABG, coronary artery bypass grafting; MI, myocardial infarction; PCI, percutaneous coronary intervention; STEMI, ST-elevation myocardial infarction; NSTEMI, non-ST-elevation myocardial infarction; MAP, mean arterial pressure; SOFA, sequential organ failure assessment.

**Figure 1:**
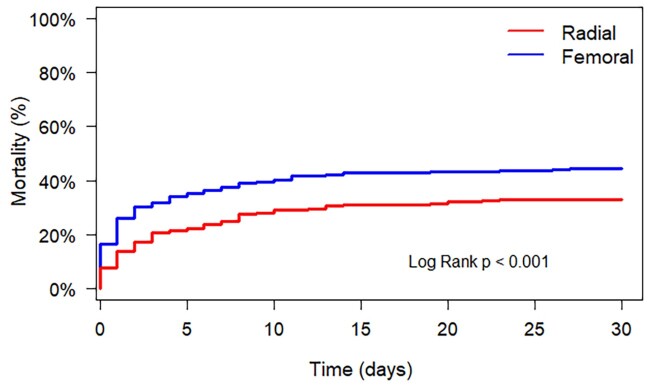
Propensity score–matched survival analysis. Propensity score matching was done on all available patient-level characteristics. This resulted in 507 matches. The survival curve of the matched patients shows a significant difference in favour of patients treated through transradial approach. Transradial access should perhaps be the preferred approach given its favourable outcomes.

Patients who underwent TFA PCI had a significantly higher unadjusted mortality rate at 30 days: 47% vs. 26% (*P* < 0.001) in TRA. This difference was also present at 1 year: 55% vs. 36% (*P* < 0.001). The difference in mortality rates was also seen in the PSM analysis: 46% vs. 33% (*P* < 0.001; see *Figure 1* and *Table 3*).

**Table 3:** Characteristic-matched cohort

	All (*N* = 1014)	Radial access (*N* = 507)	Femoral access (*N* = 507)	*P*-value
Baseline and medical history				
Year, *n* (%)				0.503
2017	47 (5)	22 (4)	25 (5)	
2018	170 (17)	93 (18)	77 (15)	
2019	264 (26)	122 (24)	142 (28)	
2020	304 (30)	155 (31)	149 (29)	
2021	229 (23)	115 (23)	114 (23)	
Age (years)	67 (58–75)	67 (58–75)	67 (59–75)	0.989
Male sex, *n* (%)	761 (75)	380 (75)	381 (75)	1.000
BMI (kg/m^2^)	26 (24–29)	26 (24–29)	26 (24–29)	0.802
Diabetes, *n*/*N* (%)	204/960 (21)	102/489 (21)	102/471 (22)	0.824
Prior coronary event, *n*/*N* (%)	256/977 (26)	133/496 (27)	123/481 (26)	0.712
Multivessel disease, *n*/*N* (%)	588/1009 (58)	291/505 (58)	297/504 (59)	0.722
Current presentation				
Resuscitated, *n*/*N* (%)	513/1009 (51)	254/505 (50)	259/504 (51)	0.776
STEMI, *n*/*N* (%)	856/1007 (85)	427/504 (85)	429/503 (85)	0.870
MAP (mmHg)	70 (57–87)	70 (59–87)	70 (54–88)	0.312
Heart rate, (b.p.m.)	84 (60–105)	82 (61–104)	85 (60–105)	0.652
SOFA score	10 (8–12)	10 (8–12)	9 (8–12)	0.489
Symptoms > 24 h, *n*/*N* (%)	137/881 (16)	72/447 (16)	65/434 (15)	0.711
Symptoms < 3 h, *n*/*N* (%)	524/881 (59)	264/447 (59)	260/434 (60)	0.851
Laboratory values				
Lactate (mmol/L)	5.2 (2.6–8.6)	5.4 (2.5–8.6)	4.9 (2.6–8.6)	0.767
Glucose (mmol/L)	12.3 (9.3–17.0)	12.3 (9.2–16.8)	12.3 (9.3–17.3)	0.639
Haemoglobin (mmol/L)	8.4 (7.4–9.3)	8.4 (7.4–9.3)	8.4 (7.4 –9.3)	0.766
eGFR (mL/min)	61 (47–74)	61 (47–75)	62 (48–73)	0.890
Hs-troponin T (peak; ng/L)	3539 (778–10 000)	3980 (879–10 000)	3190 (674–10 000)	0.223
CKMB (peak; µg/L)	232 (71–483)	257 (76–483)	198 (58–480)	0.130
Procedural details				
TIMI flow pre-PCI, *n*/*N* (%)				0.960
0/1	673/879 (76)	341/443 (67)	332/436 (77)	
2	80/879 (9)	39/443 (9)	41/436 (9)	
3	126/879 (14)	63/443 (14)	63/436 (14)	
TIMI flow post-PCI, *n*/*N* (%)				0.918
0/1	89/892 (10)	42/446 (10)	47/446 (11)	
2	69/892 (8)	34/446 (8)	35/446 (8)	
3	734/892 (82)	370/446 (83)	364/446 (82)	
Intervention in LCA, *n*/*N* (%)	648/1000 (65)		325/504 (65)	0.885
Intervention in RCA, *n*/*N* (%)	413/1000 (41)		211/504 (42)	0.763
Treatment				
Inotropes pre-PCI, *n*/*N* (%)	561/985 (57)	283/495 (57)	278/490 (57)	0.941
Intubated pre-PCI, *n*/*N* (%)	481/1009 (48)	240/504 (48)	241/505 (48)	1.000
MCS, *n*/*N* (%)	227/1005 (23)	110/502 (22)	117/503 (23)	0.663
Outcome				
30-day mortality, *n*/*N* (%)	391/1007 (39)	166 (33)	225 (45)	<0.001

Values are median (interquartile range) unless indicated otherwise. Coronary event: myocardial infarction, and/or percutaneous coronary intervention and/or coronary artery bypass grafting; resuscitated: either in or out of hospital cardiac arrest pre-PCI. Inotropes included the following agents initiated prior to PCI: noradrenaline, adrenaline, dobutamine, dopamine, and milrinone/enoximone.

BMI, body mass index; STEMI, ST-elevation myocardial infarction; MAP, mean arterial pressure; b.p.m., beats per minute; SOFA, sequential organ failure assessment; eGFR, estimated glomerular filtration rate; Hs-troponin T, high-sensitive troponin T; CKMB, creatine kinase myocardial band; TIMI, thrombolysis in myocardial infarction; LCA, left coronary artery; RCA, right coronary artery.

### Mechanical circulatory support

A total of 335 patients received MCS shortly after hospital admission. In both the TRA and the TFA group, 5% of patients (*n* = 31 and *n* = 45, respectively) were treated with MCS therapy before entering the catheterization laboratory. However, the rate of MCS initiation during the revascularization procedure was 20% in TFA patients and only 6% in TRA patients.

A sensitivity analysis after removing all patients receiving MCS before or during the PCI showed similar results (see Supplementary material online, *Table S3*). Again, the distribution between TRA and TFA treatment was approximately equal, and there were no differences in comorbidities compared with the primary analysis. Furthermore, in this sensitivity analysis, TFA patients had worse haemodynamic parameters and increased laboratory measures of hypoperfusion.

### Crossover

**Figure 2:**
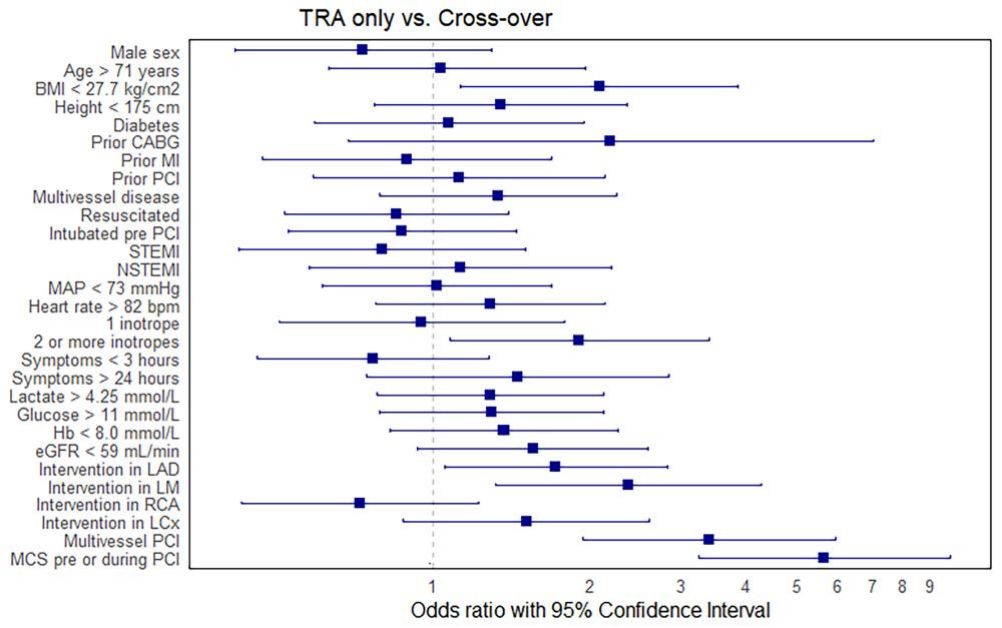
Unadjusted odds ratios for crossover. Outcomes of univariate logistic regression worth femoral-to-radial crossover as the dependent variable. The increased frequency of requiring a second access site in patients with mechanical circulatory support can be attributed, in part, to the prior utilization of the alternative access site. With regard to interventions in the left main and left anterior descending coronary artery, this might indicate difficulty of these specific procedures. The use of ≥2 vasopressors/inotropes before percutaneous coronary intervention suggests that transradial access may be impeded by excessive vasoconstriction. Cut-offs for continuous variables represent the points closest to the upper left corner from the receiver operating characteristic curve. BMI, body mass index; CABG, coronary artery bypass grafting; MI, myocardial infarction; PCI, percutaneous coronary intervention; NSTEMI, non-ST-elevation myocardial infarction; MAP, mean arterial pressure; b.p.m., beats per minute; LAD, left anterior descending coronary artery; LM, left main coronary artery; RCA, right coronary artery; LCx, left circumflex coronary artery; MCS, mechanical circulatory support; TRA, transradial access.

Of the 553 patients in whom PCI was initiated using TRA, 77 (14%) required vascular crossover from TRA to TFA. Using univariable logistic regression with a *P*-value of 0.10, 7 factors were identified to be associated with crossover (see *Figure 2* and Supplementary material online, *Table S4*). Factors most strongly predisposing crossover include a body mass index (BMI) ≤ 27.7 kg/m^2^, a history of CABG, ≥2 inotropes pre-PCI, an intervention in the left main coronary artery (LM), undergoing a multivessel PCI, and the initiation of MCS before or during the intervention.

## Discussion

This analysis in 1562 patients with AMI complicated by CS from the Dutch national NHR registry showed relevant differences in baseline characteristics and access site–specific outcomes for TRA- and TFA-treated PCI patients. Important differences in comorbidity, baseline characteristics, and mortality were identified. Specifically, TFA patients presented with worse haemodynamics, more often had a history of CABG and had a higher rate of mortality after 30 days, even after correction for potential confounders. Furthermore, 14% of patients required radial-to-femoral crossover, and this was mainly driven by the need for MCS insertion.

There are currently no data available from randomized controlled trials to clearly recommend a preferred vascular access site for PCI in AMI-CS patients. Therefore, the choice of vascular access is influenced by operators’ personal preferences depending on perceived advantages and disadvantages of TRA and TFA. By immediately accessing through the femoral artery, operators avoid the risk of having to cross over in exchange for a higher rate of bleedings and other vascular complications associated with TFA. Accessing through the radial artery results in a lower complication rate in AMI PCI in haemodynamically stable patients.^3^ However, in haemodynamically unstable patients, TRA could result in a longer time to revascularization (and haemodynamic stabilization) due to difficulties puncturing and, moreover, running the risk of having to cross over and use a second access site after all.

Even though this is not the first study to describe an AMI-CS population with regard to the vascular access site, the current manuscript adds substantial knowledge to this topic. By focusing on patient characteristics rather than on outcomes only, we were able to provide detailed insights in the incentives for choosing one or the other access approach. We have demonstrated that in this cohort, TFA patients differed significantly from TRA patients in terms of baseline and haemodynamic characteristics.

Hypotension (and subsequent accompanying measures of organ hypoperfusion) was one of the most important factors associated with choosing TRA over TFA. Mean blood pressures on admission were lower, and both lactate and creatinine levels were higher in TFA patients. In contrast to a recent study, we found that patients’ age and BMI were equally distributed in the two groups.^14^

Earlier research that focused on identifying risk factors for TRA failure revealed that higher age, female sex, short stature, and a history of CABG were independent predictors of TRA failure in AMI patients without CS.^15,16^ Of those, only having a history of CABG showed clinically relevant difference between the two groups in our study. Apparently, the other factors (age, sex, and posture) did not necessarily lead to an immediate femoral approach by operators. Noteworthy, the strongest predictor of TRA failure in regular AMI PCI is the presence of shock.^15^

The small number of patients requiring crossover limited our potential to identify factors associated with this phenomenon in a multivariate analysis. Nonetheless, initiation of MCS in the catheterization laboratory was associated with crossover. The increased frequency of requiring a second access site in patients with MCS can be attributed, in part, to the prior utilization of the alternative access site. We did find unadjusted odds ratios of 1.7 and 2.4 for interventions in the LM and the left anterior descending coronary artery (LAD), respectively. This might indicate difficulty of these specific procedures. Alternatively, significant collinearity may exist between interventions in the LM or LAD and initiation of MCS. We also found an odds ratio of 1.8 for the use of ≥2 vasopressors/inotropes before PCI which suggests that TRA may be impeded by excessive vasoconstriction.

We reported a significant temporal trend of an increased use of TRA during the study period. Even though similar trends have previously reported in other national PCI registry, important differences remain in absolute rates of TRA between different countries. Registry data from the USA showed that TRA was used in 29% of AMI-CS PCIs in 2021, which is much lower than the 47% reported in our current data. This observation becomes even more striking when considering that the severity of shock in this American population was probably lower, given the relatively low mortality rate of 20%. It is challenging to put these results in an international perspective as rates of TFA use vary widely over different countries.^14,17,18^

A sensitivity analysis leaving out all patients receiving any form of MCS was performed, as we suspected that the presence or need for a MCS device will strongly influence the choice for access site. The results of this analysis were similar to the primary analysis. This shows that the factors that determine which approach is chosen are irrespective of MCS use and thereby add robustness to the results of the primary analysis.

### Strengths and limitations

This is the first study to provide a detailed description of the differences in patient characteristics of CS patients undergoing PCI with either TRA or TFA from a large national cohort. The new insights may allow a better interpretation of other studies focusing on vascular access site for PCI in AMI-CS patients. Furthermore, the PSM analysis is the first one in its kind and provides the best available evidence for the association between mortality and access site. Additionally, the current analysis showed that TRA is achievable in a large portion of AMI-CS patients. We have attempted to retain to a homogeneous population by excluding patients who developed shock during or after PCI, even though this hardly influenced the results.

However, the current analysis has some limitations that should be acknowledged. First and foremost, the individual reasons for radial-to-femoral crossover were not known. We assume that difficulty puncturing or experiencing time pressure may have played an important role. However, crossover is perhaps more often due to peripheral vascular conditions and tortuosity than due to failed puncture of radial artery spasm.^19^ Furthermore, we were not able to differentiate between a radial access revascularization that used femoral for MCS vs. a radial access that was truly transformed into a femoral access revascularization plus MCS (true crossover). And lastly, we were not able to report details on complication rates and outcomes other than mortality as they are not a part of the national registry.

## Conclusions

This study highlights significant differences in patient characteristics between AMI-CS patients undergoing PCI via TRA or TFA. In the current cohort, femoral access was more often deployed in female patients and those with a history of CABG or a preceding cardiac arrest. Operators also deferred to TFA in patients with more pronounce haemodynamic distress or those wither a longer duration of symptoms. Nonetheless, the current study underscores the feasibility and major impact on survival of the radial approach for PCI in CS.

## Supplementary Material

zuae078_Supplementary_Data

## Data Availability

The data presented in this study were obtained from the Netherlands Heart Registration and are not openly available. Data may be provided upon request.
