# Supplementary material for: Characteristics and outcome in cardiogenic shock according to vascular access site for percutaneous coronary intervention
Source: Eur Heart J Acute Cardiovasc Care. 2024 Jun 26;13(8):615–23. doi: 10.1093/ehjacc/zuae078 (PMC11350431; doi:10.1093/ehjacc/zuae078)
Supplement: zuae078_Supplementary_Data [file zuae078_supplementary_data.zip › Supplemental material.docx]

Supplementary material

- Table S1: Baseline characteristics including patients who developed shock during or after PCI
- Table S2: Univariate and multivariate logistic regression for 30-day mortality
- Table S3: Baseline characteristics – sensitivity analyses excluding all patients treated with MCS
- Table S4: Logistic regression for cross-over

| **Supplementary TABLE S1**  Baseline characteristics including patients who developed shock during or after PCI | | | | | | |
| --- | --- | --- | --- | --- | --- | --- |
|  | | | All  N = 2045 | Radial access  N = 1013 | Femoral access  N = 1032 | P-value |
| **Baseline and medical history** | | | | | | |
| Age – years | | | 67 (58 – 75) | 67 (58 – 75) | 68 (59 – 76) | 0.331 |
| Male sex – no. (%) | | | 1509 (74) | 783 (77) | 726 (70) | <0.001 |
| BMI – kg/m^2^ | | | 26 (24 – 29) | 26 (24 – 29) | 26 (24- 29) | 0.637 |
| Height – cm | | | 175 (168 – 180) | 175 (170 – 181) | 175 (167 – 180) | 0.005 |
| Medical history – no./total no. (%) | | | | | | |
|  | *Diabetes* | | 415/1957 (21) | 189/988 (19) | 226/969 (23) | 0.023 |
|  | *Multivessel disease* | | 1220/2034 (60) | 628/1008 (62) | 592/1026 (58) | 0.034 |
|  | *Prior CABG* | | 123/2016 (6) | 28/1005 (3) | 95/1011 (9) | <0.001 |
|  | *Prior MI* | | 432/1989 (22) | 195/998 (20) | 237/991 (24) | 0.018 |
|  | *Prior PCI* | | 368/1991 (18) | 163/1000(16) | 205/991 (21) | 0.012 |
| **Current presentation** | | | | | | |
| MAP – mmHg | | | 76 (61 – 93) | 78 (65 – 94) | 73 (58 – 92) | <0.001 |
| Heart rate – bpm | | | 83 (64 – 102) | 82 (65 – 100) | 85 (62 – 103) | 0.751 |
| SOFA score | | | 10 (7 – 12) | 10 (7 – 12) | 10 (8 – 13) | 0.003 |
| No. of inotropes – n/N (%) | | | | | | |
|  | *0* | | 998/1953 (51) | 622/979 (64) | 376/974 (39) | <0.001 |
|  | *1* | | 522/1953 (27) | 197/979 (20) | 325/974 (33) | <0.001 |
|  | *2* | | 347/1953 (18) | 127/979 (13) | 220/974 (23) | <0.001 |
|  | *>=3* | | 86/1953 (4) | 33/979 (3) | 53/974 (5) | 0.026 |
| Etiology – n/N (%) | | | | | | |
|  | *STEMI* | | 1689/2032 (84) | 830/1007 (82) | 868/1025 (85) | 0.169 |
|  | *NSTEMI* | | 281/2032 (14) | 148/1007 (15) | 133/1025 (13) | 0.261 |
| Symptoms >24 hours – n/N (%) | | | 304/1757 (17) | 131/890 (15) | 173/867 (20) | 0.004 |
| Resuscitated – n/N (%) | | | 954/2031 | 382/1005 | 572/1026 | <0.001 |
| Intubated – n/N (%) | | | 908/2028 (45) | 328/1003 (33) | 580/1025 (57) | <0.001 |
| **Laboratory values** | | | | | | |
| Glucose – mmol/L | | | 12.1 (8.9 – 17.2) | 10.8 (8.2 – 15.2) | 13.5 (9.8 – 18.8) | <0.001 |
| Lactate – mmol/L | | | 5.6 (2.6 – 9.3) | 4.0 (2.1 – 7.6) | 6.6 (3.4 – 10.5) | <0.001 |
| Hemoglobine mmol/L | | | 8.4 (7.4 – 9.2) | 8.5 (7.6 – 9.3) | 8.3 (7.3 – 9.2) | 0.002 |
| eGFR – mL/min | | | 61 (48 – 76) | 65 (50 – 80) | 59 (45 – 72) | <0.001 |
| **Mechanical circulatory support** | | | | | | |
| None – n/N (%) | | | 1505/1977 (76) | 758/967 (78) | 747/1010 (74) | 0.021 |
| Before PCI – n/N (%) | | | 176/1977 (9) | 67/967 (7) | 109/1010 (11) | 0.003 |
| In cath lab, after PCI – n/N (%) | | | 244/1977 (12) | 116/967 (12) | 128/1010 (13) | 0.647 |
| **Outcome** | | | | | | |
| 30-d mortality – n/N (%) | | | 793/2032 (39) | 288/1006 (29) | 505/1026 (49) | <0.001 |
| 1-y mortality – n/N (%) | | | 617/1390 (44) | 240/386 (35) | 377/707 (53) | <0.001 |
|  | | |  | | | |
|  | | Values are median (interquartile range) unless indicated otherwise. **BMI** = body mass index; **CABG** = coronary artery bypass grafting; **MI** = myocardial infarction; **PCI** = percutaneous coronary intervention; **MAP** = mean arterial pressure; **bpm** = beat per minute; Inotropes included the following agents initiated prior to PCi: noradrenaline, adrenaline, dobutamine, dopamine, milrinon / enoximone; **STEMI** = ST-elevation myocardial infarction; **NSTEMI** = non ST-elevation myocardial infarction; **OHCA** = out of hospital cardiac arrest; **IHCA** = in hospital cardial arrest | | | | |

| **SUPPLEMENTARY TABLE S2**  Univariate and multivariate logistic regression for 30-day mortality in all patients with shock pre PCI | | | | | | | | |
| --- | --- | --- | --- | --- | --- | --- | --- | --- |
|  | | | **OR univariate** | **95% CI** | **p-value** | **OR multivariate** | **95% CI** | **p-value** |
| Year *(reference 2021)* | | |  |  |  |  |  |  |
|  | 2020 | | 0.928 | 0.697 – 1.235 | 0.609 |  |  |  |
|  | 2019 | | 1.088 | 0.813 – 1.457 | 0.570 |  |  |  |
|  | 2018 | | 1.142 | 0.832 – 1.567 | 0.411 |  |  |  |
|  | 2017 | | 1.343 | 0.813 – 2.218 | 0.246 |  |  |  |
| Age | | | 1.028 | 1.019 – 1.037 | <0.001 | 1.046 | 1.484 – 1.033 | <0.001 |
| Male sex | | | 0.988 | 0.784 – 1.245 | 0.917 |  |  |  |
| BMI | | | 1.020 | 0.991 – 1.049 | 0.160 |  |  |  |
| Diabetes Mellitus | | | 1.742 | 1.362 – 2.227 | <0.001 |  |  |  |
| Prior coronary event | | | 1.286 | 1.025 – 1.614 | 0.030 |  |  |  |
| Multivessel disease | | | 1.837 | 1.490 – 2.264 | <0.001 | 1.484 | 1.133 – 1.944 | 0.004 |
| Cardiac arrest | | | 1.782 | 1.453 – 2.186 | <0.001 | 1.768 | 1.276 – 2.449 | <0.001 |
| STEMI *(vs. NSTEMI)* | | | 1.612 | 1.201 – 2.163 | 0.001 |  |  |  |
| MAP | | | 0.992 | 0.987 – 0.997 | 0.002 | 0.992 | 0.985 – 0.998 | 0.010 |
| Heart rate | | | 1.007 | 1.004 – 1.011 | <0.001 | 1.008 | 1.003 - 1.014 | 0.002 |
| SOFA | | | 1.147 | 1.056 – 1.247 | 0.006 |  |  |  |
| Inotropes pre (*yes/no)* | | | 2.335 | 1.893 – 2.878 | <0.001 | 1.588 | 1.167 – 2.159 | 0.003 |
| Duration of symptoms | | |  |  |  |  |  |  |
|  | *<3 hours* | | 0.819 | 0.664 – 1.012 | 0.064 |  |  |  |
|  | *>24 hours* | | 1.858 | 1.415 – 2.439 | <0.001 | 1.719 | 1.164 – 2.539 | 0.007 |
| Lactate | | | 1.153 | 1.123 – 1.184 | <0.001 | 1.084 | 1.041 – 1.129 | <0.001 |
| Glucose | | | 1.088 | 1.068 – 1.107 | <0.001 | 1.049 | 1.021 – 1.078 | <0.001 |
| Hemoglobin | | | 0.845 | 0.786 – 0.910 | <0.001 | 0.872 | 0.789 – 0.964 | 0.007 |
| eGFR | | | 0.979 | 0.973 – 0.984 | <0.001 | 0.991 | 0.985 – 0.997 | 0.005 |
| MCS | | | 2.548 | 2.008 – 3.235 | <0.001 | 2.407 | 1.758 – 3.294 | <0.001 |
| TIMI-flow after PCI (reference: 3) | | |  |  |  |  |  |  |
|  | *TIMI_flow 2* | | 1.676 | 1.150 – 2.443 | 0.007 | 1.863 | 1.162 – 2.989 | 0.010 |
|  | *TIMI-flow 1* | | 4.458 | 2.336 – 8.507 | <0.001 | 4.468 | 1.842 – 10.835 | 0.002 |
|  | *TIMI-flow 0* | | 5.901 | 3.531 – 9.864 | <0.001 | 4.850 | 2.711 – 8.678 | <0.001 |
| Femoral access | | | 2.539 | 2.060 – 3.130 | <0.001 | 1.727 | 1.322 – 2.258 | <0.001 |
|  | | **BMI** = body mass index, per kg/m^2^; Prior coronary event = prior myocardial infarction or percutaneous coronary intervention or coronary artery bypass grafting; **STEMI** = ST-elevation myocardial infarction; **MAP** = Mean arterial pressure, per mmHg; **SOFA** = Sequential organ failure assessment, per point; **Lactate** per mmol/L; **Glucose** per mmol/L; **Hemoglobin** per mmol/L; **eGFR** per mL/min; **MCS** = mechanical circulatory support; **TIMI** = thrombolysis in myocardial infarction flow grade | | | | | | |

| **Supplementary TABLE S3**  Baseline characteristics of patient with shock pre PCI – sensitivity analyses excluding all patients treated with MCS | | | | | | | | | | |  | |  |  |  |  |
| --- | --- | --- | --- | --- | --- | --- | --- | --- | --- | --- | --- | --- | --- | --- | --- | --- |
|  | | | | | All  N = 1178 | | | Radial access  N = 548 | Femoral access  N = 630 | | P-value | |  |  |  |  |
| **Baseline and medical history** | | | | | | | | | | | | |  |  |  |  |
| Age – years | | | | | 68 (58 – 76) | | | 67 (58 – 75) | 69 (60 – 76) | | 0.133 | |  |  |  |  |
| Male sex – no. (%) | | | | | 854 (72) | | | 425 (77) | 429 (68) | | <0.001 | |  |  |  |  |
| BMI – kg/m^2^ | | | | | 26 (24 – 29) | | | 26 (24 – 29) | 26 (24 – 29) | | 0.767 | |  |  |  |  |
| Height – cm | | | | | 175 (168 – 180) | | | 175 (170 – 180) | 175 (167 – 180) | | 0.038 | |  |  |  |  |
| Medical history – no./total no. (%) | | | | | | | | | | | | |  |  |  |  |
|  | *Diabetes* | | | | 233/1117 (21) | | | 96/533 (18) | 137/584 (23) | | 0.025 | |  |  |  |  |
|  | *Multivessel disease* | | | | 663/1171 (57) | | | 326/545 (60) | 337/626 (54) | | 0.039 | |  |  |  |  |
|  | *Prior CABG* | | | | 75/1156 (6) | | | 12/543 (2) | 63/613 (10) | | <0.001 | |  |  |  |  |
|  | *Prior MI* | | | | 241/1138 (21) | | | 103/538 (19) | 138/600 (23) | | 0.112 | |  |  |  |  |
|  | *Prior PCI* | | | | 202/1140 (18) | | | 86/538 (16) | 116/602 (19) | | 0.147 | |  |  |  |  |
| **Current presentation** | | | | | | | | | | | | |  |  |  |  |
| MAP – mmHg | | | | | 72 (59 – 90) | | | 73 (61 – 93) | 70 (57 – 88) | | 0.001 | |  |  |  |  |
| Heart rate – bpm | | | | | 81 (60 – 101) | | | 80 (61 – 103) | 82 (56 – 101) | | 0.480 | |  |  |  |  |
| SOFA score | | | | | 10 (7 – 12) | | | 10 (7 – 12) | 10 (8 – 13) | | 0.579 | |  |  |  |  |
| No. of inotropes – n/N (%) | | | | | | | | | | | | |  |  |  |  |
|  | *0* | | | | 511/1124 (45) | | | 304/530 (57) | 207/594 (35) | | <0.001 | |  |  |  |  |
|  | *1* | | | | 345/1124(31) | | | 133/530 (25) | 212/594 (36) | | <0.001 | |  |  |  |  |
|  | *2* | | | | 222/1124(20) | | | 75/597 (14) | 147/594 (25) | | <0.001 | |  |  |  |  |
|  | *>=3* | | | | 46/1124 (4) | | | 18/530 (3) | 28/594 (5) | | 0.266 | |  |  |  |  |
| Etiology – n/N (%) | | | | | | | | | | | | |  |  |  |  |
|  | *STEMI* | | | | 1003/1172 (86) | | | 463/545 (85) | 540/627 (86) | | 0.569 | |  |  |  |  |
|  | *NSTEMI* | | | | 149/1172 (13) | | | 71/545 (13) | 78/627 (12) | | 0.763 | |  |  |  |  |
| Symptoms >24 hours – n/N (%) | | | | | 159/1020 (16) | | | 66/492 (13) | 93/528 (18) | | 0.090 | |  |  |  |  |
| Resuscitated – n/N (%) | | | | | 597/1173 (51) | | | 244/547 (45) | 353/626 (56) | | <0.001 | |  |  |  |  |
| Intubated – n/N (%) | | | | | 500/1076 (46) | | | 176/487 (36) | 324/589 (55) | | <0.001 | |  |  |  |  |
| **Laboratory values** | | | | | | | | | | | | |  |  |  |  |
| Glucose – mmol/L | | | | | 12.0 (9.0 – 17.0) | | | 11.0 (8.5 – 15.0) | 13.3 (9.6 – 18.8) | | <0.001 | |  |  |  |  |
| Lactate – mmol/L | | | | | 5.6 (2.8 – 9.2) | | | 4.0 (2.3 – 7.7) | 6.7 (3.5 – 10.6) | | <0.001 | |  |  |  |  |
| Hemoglobine mmol/L | | | | | 8.4 (7.4 – 9.2) | | | 8.6 (7.6 – 9.3) | 8.2 (7.3 – 9.1) | | <0.001 | |  |  |  |  |
| eGFR – mL/min | | | | | 61 (48 – 75) | | | 64 (52 – 79) | 58 (86 – 128) | | <0.001 | |  |  |  |  |
| **Outcome** | | | | | | | | | | | | |  |  |  |  |
| 30-d mortality – n/N (%) | | | | | 436/1171 (37) | | | 141/544 (26) | 295/627 (47) | | <0.001 | |  |  |  |  |
| 1-y mortality – n/N (%) | | | | | 351/825 (43) | | | 127/382 (33) | 224/443 (51) | | <0.001 | |  |  |  |  |
|  | | | | |  | | | | | | | |  |  |  |  |
|  | | Values are median (interquartile range) unless indicated otherwise. **BMI** = body mass index; **CABG** = coronary artery bypass grafting; **MI** = myocardial infarction; **PCI** = percutaneous coronary intervention; **MAP** = mean arterial pressure; **bpm** = beat per minute; Inotropes included the following agents initiated prior to PCi: noradrenaline, adrenaline, dobutamine, dopamine, milrinon / enoximone; **STEMI** = ST-elevation myocardial infarction; **NSTEMI** = non ST-elevation myocardial infarction; **OHCA** = out of hospital cardiac arrest; **IHCA** = in hospital cardial arrest | | | | | | | | | | |  |  |  |  |
|  | | | | | | | | | | | | | | | | |
|  | | | | | |  |  |  |  |  |  |  |  |  |  |  |
| **Supplementary Table S4**  Logistic regression for cross-over | | | | | | | | | | | |  |  |  |  |  |
|  | | | | | | **OR univariate** | | **95% CI** | | | | **p-value** | |  |  |  |
| Age > 71 | | | | | | 1.032 | | | 0.629-1.962 | | | 0.900 | |  |  |  |
| Male sex | | | | | | 0.733 | | | 0.416 – 1.291 | | | 0.282 | |  |  |  |
| BMI ≤ 27.7 kg/m^2^ | | | | | | 2.083 | | | 1.126-3.852 | | | 0.019 | |  |  |  |
| Height ≤175 cm | | | | | | 1.351 | | | 0.773-2.361 | | | 0.288 | |  |  |  |
| Diabetes | | | | | | 1.071 | | | 0.590-1.944 | | | 0.821 | |  |  |  |
| Prior CABG | | | | | | 2.187 | | | 0.687-6.974 | | | 0.185 | |  |  |  |
| Prior MI | | | | | | 0.890 | | | 0.469-1.688 | | | 0.721 | |  |  |  |
| Prior PCI | | | | | | 1.120 | | | 0.587-2.139 | | | 0.731 | |  |  |  |
| Multivessel disease | | | | | | 1.333 | | | 0.789-2.254 | | | 0.283 | |  |  |  |
| Resuscitated | | | | | | 0.851 | | | 0.519-1.394 | | | 0.522 | |  |  |  |
| Intubated pre PCI | | | | | | 0.871 | | | 0.527-1.440 | | | 0.591 | |  |  |  |
| STEMI | | | | | | 0.798 | | | 0.424-1.500 | | | 0.483 | |  |  |  |
| NSTEMI | | | | | | 1.130 | | | 0.579-2.203 | | | 0.721 | |  |  |  |
| MAP ≤73mmHg | | | | | | 1.017 | | | 0.614-1.684 | | | 0.947 | |  |  |  |
| Heart rate >82 bpm | | | | | | 1.289 | | | 0.776-2.139 | | | 0.326 | |  |  |  |
| Inotropes pre PCI | | | | | |  | | |  | | |  | |  |  |  |
|  | *1* | | | | | 0.951 | | | 0.506-1.786 | | | 0.875 | |  |  |  |
|  | *≥ 2* | | | | | 1.905 | | | 1.074-3.377 | | | 0.027 | |  |  |  |
| Duration of symptoms | | | | | |  | | |  | | |  | |  |  |  |
|  | *< 3 hours* | | | | | 0.766 | | | 0.460-1.276 | | | 0.305 | |  |  |  |
|  | *>24 hours* | | | | | 1.452 | | | 0.745-2.831 | | | 0.272 | |  |  |  |
| Lactate >4.25 – mmol/L | | | | | | 1.287 | | | 0.778-2.129 | | | 0.326 | |  |  |  |
| Glucose >11 – mmol/L | | | | | | 1.294 | | | 0.789-2.123 | | | 0.307 | |  |  |  |
| Hemoglobine ≤8.0 – mmol/L | | | | | | 1.367 | | | 0.826-2.262 | | | 0.224 | |  |  |  |
| eGFR ≤ 59 mL/min | | | | | | 1.552 | | | 0.933-2.582 | | | 0.091 | |  |  |  |
| Treated vessel | | | | | |  | | |  | | |  | |  |  |  |
|  | *LAD* | | | | | 1.720 | | | 1.053-2.809 | | | 0.030 | |  |  |  |
|  | *LM* | | | | | 2.365 | | | 1.315-4.251 | | | 0.004 | |  |  |  |
|  | *RCA* | | | | | 0.722 | | | 0.428-1.221 | | | 0.224 | |  |  |  |
|  | *RCX* | | | | | 1.508 | | | 0.876-2.596 | | | 0.138 | |  |  |  |
| Multivessel PCI | | | | | | 3.383 | | | 1.937-5.907 | | | <0.001 | |  |  |  |
| MCS pre or during PCI | | | | | | 5.623 | | | 3.229-9.791 | | | <0.001 | |  |  |  |
|  | | | | | **BMI** = body mass index; **CABG** = coronary artery bypass grafting; **MI** = myocardial infarction; **PCI** = percutaneous coronary intervention; **STEMI** = ST-elevation myocardial infarction; **MAP** = Mean arterial pressure, per mmHg; **SOFA** = Sequential organ failure assessment, per point; **eGFR** = estimated glomerular filtration rate; **LAD** = left anterior descending; **LM** = left main; **RCA** = right coronary artery; **RCX** = right circumflex; **MCS** = mechanical circulatory support; **TIMI** = thrombolysis in myocardial infarction flow grade | | | | | | | | |  |  |  |
